# The Queen Square cognitive assessment screen (Q-CAS): normative data and validation in acute stroke

**DOI:** 10.1007/s00415-026-14016-4

**Published:** 2026-07-20

**Authors:** Edgar Chan, Maria del Rocio Hidalgo Mas, Adrian So, Robert Simister, David J. Werring, Parashkev Nachev, Lisa Cipolotti

**Affiliations:** 1 Department of Neuropsychology, National Hospital for Neurology & Neurosurgery, UCLH, Queen Square, Box 37, London, WC1N 3BG UK; 2 UCL Stroke Research Centre, Department of Translational Neuroscience and Stroke, UCL Queen Square Institute of Neurology, London, UK; 3 University College London, London, UK; 4 Department of Translational Neuroscience and Stroke, UCL Queen Square Institute of Neurology, London, UK

**Keywords:** Cognition, Neuropsychology, Screening, Executive functions, Mood

## Abstract

**Background:**

Accurate and comprehensive assessment of cognition after stroke is crucial for diagnosis and rehabilitation. However, detailed assessment by a Neuropsychologist is often not feasible or practical. Although many screening tools exist, none fulfill three critical criteria: 1) assess all stroke-relevant domains, 2) consider demographic variables of gender, age, education level, and whether English is a first or second language, and 3) is brief and can be easily administered.

**Methods:**

The Q-CAS was developed by adapting well-validated neuropsychological tests. Cognitive domains include verbal/spatial memory, naming, perception, attention, executive functions, processing speed, and mood. We administered the Q-CAS to healthy individuals and patients with acute mild-to-moderate stroke, and examined aspects of reliability, convergent and divergent validity, and diagnostic accuracy against reference-standard neuropsychological testing.

**Results:**

648 healthy individuals and 150 patients with acute stroke were assessed. The mean completion time was 9 min for healthy individuals and 15 min for stroke patients. All Q-CAS subtests demonstrated clinically acceptable convergent and divergent validity against neuropsychological tests. ROC curve analyses showed good discrimination for all cognitive domains and were comparable for left- and right-hemisphere lesions. Using a novel simple scoring method which incorporates demographic variables, executive function, speed, and memory showed the best sensitivity (> 0.8), whereas perception and attention showed the best specificity (> 0.8).

**Conclusion:**

The Q-CAS is a brief cognitive screening tool assessing six core cognitive domains and mood. Validated in acute stroke, it demonstrates good discrimination across domains and offers a practical first-line approach that mitigates limitations of existing tools while balancing brevity and thoroughness.

**Supplementary Information:**

The online version contains supplementary material available at 10.1007/s00415-026-14016-4.

## Introduction

The accurate assessment of cognition in suspected and confirmed neurological conditions plays a key role in diagnostic and rehabilitation neurology. Neuropsychological assessment by a qualified neuropsychologist remains the gold standard. However, briefer cognitive screening tools are a pragmatic alternative in both clinical and research settings. Screening tools increase accessibility and usability as a first-line approach, ideally flagging patients who require more comprehensive expert assessment rather than replacing it [1]. This is particularly pertinent for neurological conditions where cognitive impairments are common sequelae, for example, in stroke.

Stroke is the third leading cause of death and disability worldwide [2], and cognitive impairment has been reported in approximately 60% of stroke survivors the first year after stroke [3]. Impaired cognition after stroke is associated with increased hospital length of stay [4], poorer long-term functional outcomes [5, 6], decreased quality of life, and increased carer burden [7]. Poor understanding of the cognitive impact of stroke can lead to long-term unmet needs and emotional distress [8], particularly for those of ethnic minorities or low socioeconomic status [9].

Cognitive screening is recommended for all patients following stroke across national and international guidelines [3, 10]. Although many screening tools are used, none has been shown to be optimally fit-for-purpose for the acute stroke population. This is because most tools are developed for detection of MCI and dementia, and therefore heavily biased toward the memory domain. Of the available screens, the MoCA is frequently used due to its combination of reasonable test accuracy with brevity [11, 12]. However, our previous work suggested that the MoCA produces too many false negatives as it does not sufficiently cover some key stroke-relevant domains, such as non-verbal memory, executive functions, and processing speed [13], and that it is less sensitive to right-hemisphere stroke deficits [14]. The Oxford Cognitive Screen (OCS) has been developed as a stroke-specific screen aiming to detect stroke-relevant domain-level impairment [15]. Like the MoCA, it does not assess non-verbal memory or processing speed, and it only uses a variant of the Trail Making Test to assess executive functions, a test shown to be nonspecific to frontal lesions [16]. A recent review found the evidence for the diagnostic accuracy and utility of the OCS for post-stroke cognitive syndromes very low [10].

In addition to poor coverage of some cognitive domains, mood is another domain that is missed from existing screening tools, even though it is considered an essential part of any comprehensive neuropsychological assessment [17]. Mood changes are very common after stroke and are associated with impaired cognitive functions and poorer functional outcomes [18]. It is well recognized that mood changes, such as depression and anxiety, often lead to alterations in attention, memory, and executive function and can mimic or exacerbate cognitive impairment [19]. Embedding of a mood screen into a cognitive screening tool ensures routine assessment, as recommended by guidelines [17], and allows coherent analysis between mood and cognitive functioning within the one measure.

One other common pitfall of existing cognitive screening tools is the use of single cut-off scores. The reductionist approach, although undeniably useful in its simplicity, falsely treats cognition as a unitary concept and ignores relevant factors, such as age, education, and cultural background. It is clear from the neuropsychology literature that demographic factors like age and education significantly impact performance on cognitive tests, particularly when assessing executive function and processing speed [20, 21]. Indeed, validation studies of screening tools such as the MoCA and MMSE have also shown clear demographic effects on test performance [22, 23]. Nevertheless, the use of normative tables for adjusted cutoffs is rarely applied in clinical practice. Similarly, although screening tests often have alternative versions in different languages, in practice, clinicians and therapists often prefer the original English version due to familiarity. Demographic factors are often only briefly considered in standardization studies, which may rely on small or unrepresentative samples. Integrating them from the outset could make them inherent to how the screen is used, while still balancing accuracy with brevity.

Here, we present a new cognitive screening tool, the Queen Square Cognitive Assessment Screen (Q-CAS), which covers six cognitive domains and mood. We collected and presented normative data from a large sample of healthy adults with a broad range of age, education level, and cultural background. We assessed individuals whose first language was English as well as those whose first language was not English, so that normative data could be used for both types of patients. We validated the screen against standardized neuropsychological assessment in a sample of patients with acute mild-to-moderate stroke. We examined the impact of demographic factors and mood on performance across domains. Finally, we presented a novel way in which the normative data could be practically incorporated into scoring for use in clinical and research settings.

## Methods

### Material

#### Q-CAS development

The Queen Square Cognitive Assessment Screen (Q-CAS) is a cognitive screening tool based on principles of neuropsychological assessment developed and used in the Department of Neuropsychology at the National Hospital for Neurology and Neurosurgery [24] covering six cognitive domains: memory, language, perception, attention, frontal executive functions, and speed of information processing. In addition, it also includes a mood screen. The Q-CAS was developed through an iterative, clinician‑led process drawing on decades of multidisciplinary experience within the stroke units at the National Hospital, Queen Square. Preparatory work included structured observations of cognitive presentations on the wards, review of existing screening limitations, and facilitated discussions with the MDT. Consensus was achieved through repeated expert meetings and pilot testing on the units, ensuring that the tool reflected real‑world clinical priorities, feasibility, and the cognitive profiles most encountered after stroke.

For simplicity and accessibility, the Q-CAS is presented on a double-sided A4 sheet and requires only a pen/pencil to administer. The subtests included in the Q-CAS and its scoring method is described in Table 1. A spatial object–location memory task was chosen, in addition to standard verbal recall, as object–location binding is thought to be hippocampal-dependent [25] and may help to differentiate memory deficits secondary to AD pathology from those due to other pathologies. Executive functions subtests selected were chosen as they have been shown to be sensitive and specific to detecting frontal dysfunction, and to balance dominant and non-dominant frontal functions [26]. Three brief measures of processing speed were selected to span a range of difficulty and to ensure that visuomotor deficits did not limit assessment. Items were intentionally designed to be relatively challenging in order to optimize detection of subtle deficits in patients with mild-to-moderate stroke, where impairments are most likely to be overlooked.

**Table 1:** Q-CAS subtests and their scoring system

Cognitive domain	Subtest	Description	Scoring
Memory (/21)	Orientation	Current month, place, D.O.B	0–3
	Verbal recall	Name and remember 6-items presented in a spatial array. Recall items at the end of the assessment	0–6
	Verbal recognition	If unable to recall, three-item multiple-choice recognition is given	0–6
	Spatial location recall	Asked to recall the location of the items in the spatial array	0–6
Language (/6)	Picture naming	Naming of 6-items graded in familiarity	0–6
Visuo-perception (/6)	Incomplete numbers	Identify 3 visually-degraded numbers	0–3
	Star counting	Count the number of stars in 3 spatial arrays	0–3
Attention (/8)	Forwards digit span	Repeat number strings of increasing length from 3–6 digits	0–4
	Backwards digit span	Repeat number strings backwards of increasing length from 2–5 digits	0–4
Executive function	Motor tapping	Finger tapping in a pattern either congruent or incongruent to the examiner or requiring inhibition according to specific rules	0–3
	Phonemic fluency	Generate as many words as possible in 1 min beginning with the letter A	No. of correct words minus no. of errors
	Design fluency	Generate as many abstract designs as possible in 1 min	No. of correct designs minus no. of errors
Processing speed	Months backwards	Recite the months of the year backwards as quickly as possible	Time taken in seconds plus no. of errors
	Counting 3 s	Counting the number of 3s on a two-row number string	Time taken in seconds plus no. of errors
	Canceling 2 s	Cross out the number of 2s on a two-row number string	Time taken in seconds plus no. of errors
Mood (/20)	Depression score	Rate current feelings of low mood from 0–10	0–10
	Anxiety score	Rate current feelings of worry from 0–10	0–10

#### Validation

To validate the Q-CAS, standardized neuropsychological tests were selected which mapped onto the same cognitive domain designed to be assessed by the different Q-CAS subtests. All patients who were administered the validation battery completed as many tasks as possible based on clinical judgment. The battery took approximately 60 min to complete. Performance on tests was scored and evaluated against normative data in published manuals. Scores at or below the 5th percentile, or it's scaled score equivalent, were generally classified as impaired. Exceptions include the revised Graded Naming Test [27], where impairment was defined as performance at or below the 2nd percentile as per the new norms. For orientation, a score of 3 or below out of 5 was considered impaired. In the mood assessments, scores of 2 or higher on both the Generalized Anxiety Disorder 2-item (GAD-2) and the Patient Health Questionnaire-2 (PHQ-2) was classified as significant mood disturbance [28, 29]. Cognitive impairment in any given domain was determined as impaired performance on at least one task within that domain.

### Participants and procedure

#### Healthy controls

Participants were recruited via written notices, word-of-mouth, and direct invitation, in and around NHNN and UCLH, a central London hospital within a diverse urban environment with a relatively high proportion of residents from either white 'other' ethnic groups (white but not British) or from Black, Asian, mixed, or other ethnic groups. A deliberate effort was made to recruit patients across a range of ages, educational, and cultural background with broad targets set for each to ensure balanced representation. Participants were excluded from participation if they had a known psychiatric, neurological, or neurodegenerative condition, or if they could not understand enough English to consent or complete the Q-CAS. For each participant, we recorded their age, whether English was their first language (EFL) or not (ESL), place of birth, handedness, years of education, and occupation. Following informed consent, assessment was conducted in a quiet and convenient environment. A cohort of healthy control participants were retested on the Q-CAS between 1 and 2 months to examine test–retest reliability.

#### Patients with acute stroke

Patients were recruited from the Hyper-Acute Stroke Unit and the Acute Stroke Unit at the NHNN between June 2022 and August 2025. Inclusion criteria were (1) aged 18 or above, (2) first-ever ischemic stroke and/or stroke due to intracerebral hemorrhage (ICH) with magnetic resonance imaging (MRI) confirmation, (3) able to give informed consent in English (4) alert and able to complete at least 50% of the assessment. Exclusion criteria were (1) presence of premorbid psychiatric, neurological, or neurodegenerative conditions, and (2) presence of other medical or physical conditions precluding participation in the assessment. For each patient, we recorded their age, first language, place of birth, handedness, years of education, and occupation. In addition, stroke type, severity (i.e., NIHSS score), lesion lateralization, and days since stroke from time of assessment were collected via the patient’s electronic records EpicCare. In addition, Limb Strength Assessment (LSA) scores were also collated from records as a divergent validity measure. The LSA was completed by therapists as part of routine clinical care during a patient’s admission on the ward.

After obtaining informed consent, patients either completed the Q-CAS only, or both the Q-CAS and validation battery during the validation phase of the study. The assessment was conducted by either a Clinical Neuropsychologist or a trained administrator under supervision. The administration order of the Q-CAS and validation battery was counterbalanced on an ABAB basis for each patient. Tests were largely administered at bedside and completed in a single session.

Based on data from previous validation studies [30, 31], a target patient sample size ranging between 68 and 154 was required to achieve 80% power at a 0.05 significance level, assuming a clinically acceptable correlation between 0.20 and 0.30.

The study was approved by The NHNN and Institute of Neurology Joint Research Ethics Committee and conducted in accordance with the Declaration of Helsinki. Written informed consent was gained for all participants.

### Statistical analyses

To examine the relationship between demographic factors on performance across the Q-CAS domains and subtests, we ran separate two-tailed correlations; we used Pearson’s correlation coefficient (r) for normally distributed data and Spearman’s rho (ρ) when assumptions were violated. Q-CAS domain scores were the combined raw score of the subtests within that domain.

#### Reliability

Test–retest reliability was assessed using the Intra-class Correlation Coefficient (ICC) with a single-measure, absolute agreement model (ICC2). ICCs were calculated separately for each subtest and for the sum of all scores (Total score). ICC values were interpreted as follows: poor (< 0.5), moderate (0.5–0.75), and good (> 0.75). Subtests with very restricted score variability resulted in ICCs that could not be computed.

#### Convergent and divergent validity

Q-CAS subtest convergent validity was assessed by comparing performance on subtests against the standardized neuropsychological tests that map on to the same cognitive domains. A correlation coefficient > 0.30 was considered adequate, and > 0.70 very good [32].

Q-CAS subtest divergent validity was assessed by correlating subtest performance with the Lower Limb Strength Assessment (LSA) score. Limb strength was assessed using the MRC grading scale (0–5) across six lower limb movements (max = 30), with higher scores indicated greater motor strength. In line with other validation studies, a correlation coefficient < 0.30 was considered acceptable [31].

#### Diagnostic accuracy of the Q-CAS

Patient performance on each cognitive domain was compared with corresponding domain impairment on neuropsychology tests. An AUC of ≥ 0.7 was considered to indicate acceptable discrimination, and ≥ 0.8 to represent excellent discrimination [33]. Analyses were conducted for the whole stroke sample and by hemisphere; differences between left- and right-hemisphere stroke performance in model performance (AUC) were assessed using nonparametric bootstrapping (10,000 iterations), with two-sided p values and 95% confidence intervals (p < 0.05).

Domain cut-offs were derived using a novel brief demographic-adjusted approach: for subtests with significant associations with age, YOE, or first language (Spearman’s ρ > 0.30, p < 0.05), Maximally Selected Rank Statistics (MSRS) was applied to identify the optimal dichotomization point for age and YOE, and EFL vs ESL for First Language. Impairment was defined as the 10th percentile within groups (or full sample if subtests were not significantly associated with demographic factors). Domain impairment was defined as any subtest impaired in that domain. Classification was validated against neuropsychology using sensitivity, specificity, PPV, and NPV.

## Results

### Normative sample

In total, 648 healthy control participants were recruited to form the normative dataset. The sample characteristics are described in Table 2. Participant ages ranged from 18 to 94 with 6–24 years of formal education. In keeping with our recruitment aim of a diverse sample, 31% of participants were of non-White ethnicity, 44% not born in the UK, and 37% whose first language was not English.

**Table 2:** Demographic and clinical characteristics of the normative and stroke sample

	Normative sample *n* = 648	Stroke patients *n* = 150
Characteristic	*n* (%)	*n* (%)
*Age (years)* 18–29/30–39/40–49 50–59/60–69/70 +	91 (14%)/93 (14%)/110 (17%) 112 (17%)/123 (19%)/119 (18%)	6 (4%)/8 (5%)/12 (8%) 37 (25%)/31 (21%)/56 (37%)
*Education (years)* ≤ 11/12–16/≥ 17	147 (23%)/323 (50%)/178 (27%)	55 (38%)/73 (51%)/16 (11%)
*Ethnicity* Asian/Black/Mixed/White/Others (e.g., Arab)	96 (15%)/61 (9%)/7(1%) 446 (69%)/38 (6%)	20 (13%)/30 (20%)/1 (1%) 79 (53%)/20 (13%)
*First language* English/Non-English	410 (63%)/238 (37%)	104 (70%)/46(30%)
*Place of Birth* UK/Non-UK	364 (56%)/284 (44%)	85 (57%)/65 (43%)
*Gender* Male/Female	279 (43%)/369 (57%)	101 (67%)/49 (33%)
Handedness Left/Right	33 (5%)/615 (95%)	21 (14%)/129 (86%)
Days since stroke (Median [IQR])	–	5 [2–8]
*Stroke type* Ischemic/Intracerebral Hemorrhage/Both	–	116 (77%)/28 (19%)/6 (4%)
*Stroke laterality* Left/Right/Both	–	63 (42%)/69 (46%)/18 (12%)
NIHSS score (median [IQR])	–	4 [2–8]

The average time taken to complete the Q-CAS was 9 min with a range of 9–11 min. The performance of the whole normative sample across subtests is summarized in Table 3. As none of the variables were normally distributed, which is typical for brief screening measures, the data are presented as median and interquartile ranges (IQR).

**Table 3:** Performance on the Q-CAS subtests for the normative and stroke sample

		Normative sample							Stroke patients			
Cognitive domain	Q-CAS subtest	*n*	10th %ile	Min	Max	Median [IQR]	Missing (n)	Missing domain (n/%)	*n*	Median [IQR]	Missing (n)	Missing domain (n/%)
Memory	Orientation	648	3	3	3	3 [3–3]	0	0	150	3 [3–3]	0	0
	Verbal Recall	648	2	0	6	4 [3–5]	0		134	3 [1–4]	16	
	Verbal Recognition	648	6	2	6	6 [6–6]	0		134	6 [5, 6]	16	
	Spatial Location Recall	648	6	0	6	6 [6–6]	0		136	6 [4–6]	14	
Language	Picture Naming	648	3	1	6	6 [5, 6]	0	0	142	4 [3–6]	8	8 (5%)
Perception	Incomplete Numbers Star Counting	648 648	2 3	0 1	3 3	3 [3–3] 3 [3–3]	0 0	0	149 149	3 [2, 3] 3 [2, 3]	1 1	1 (< 1%)
Attention	Forward Digit Span Backwards Digit Span	648 648	3 2	0 0	4 4	4 [3, 4] 3 [2–4]	0 0	0	149 149	3 [3, 4] 2 [2, 3]	1 1	1 (< 1%)
Executive Function	Motor Tapping Phonemic Fluency	648 648	3 4	0 −1	3 32	3 [3–3] 10 [6–13]	0 0	0	146 144	3 [1–3] 5 [2–8]	4 6	1 (< 1%)
	Design Fluency	644	4	−30	60	9 [6–12]	4		110	2 [0–5]	40	
Processing speed	Months Backwards Counting 3 s	644 648	23 17	5 2	67 29	13 [10–17] 11 [9–14]	4 0	0	117 138	18 [14–25] 18 [14–25]	33 12	7 (5%)
	Cancelling 2 s	645	18	7	39	13 [11–25]	3		119	22 [17–28]	31	
Mood	Depression Score	647	6	0	10	2 [0–4]	1	1 (< 1%)	93*	4 [1–7]	0	0
	Anxiety Score	647	7	0	10	2 [0–5]	1		93*	5 [2–7]	0	

^*^The mood subtest was introduced later in the validation process and was only administered to 93 patients

Performance was at ceiling only for orientation (i.e., all participants scored the maximum points), with good range of scores for all other subtests. Missing data was very low with less than 1% noncompletion for Design Fluency, Months Backwards, and Canceling 2 s subtests, either due to English proficiency or dominant-hand non-use. Only one participant declined to complete the Mood subtest.

Correlation analyses showed that older age was associated with poorer memory, perception, and speed; more education with better memory, attention, executive function, and speed; and English as a second language with poorer language, attention, and executive function (see Table 4 and S1a for subtest correlations). Sex or mood was not correlated with any cognitive domains.

**Table 4:** Correlation table showing Spearman’s rho coefficients between the Q-CAS domain performance and demographic factors and mood for healthy control participants and patients (Bold, p < 0.05)

		Memory	Language	Perception	Attention	Executive	Speed
Healthy controls	Age	−0.39**	0.04	−0.08*	−0.05	0.01	0.24**
	YOE	0.22**	−0.04	0.07	0.15**	0.19**	−0.16**
	FL	0.06	−0.59**	−0.04	−0.16**	−0.28**	−0.04
	Sex	0.04	−0.06	0	0.04	−0.01	−0.01
	Mood	0.05	0.04	−0.05	−0.04	0.02	0.04
Stroke patients	Age	−0.25*	−0.26*	−0.12	0.01	−0.13	0.20*
	YOE	0.15	0.26*	0.14	0.24*	0.37**	−0.28*
	FL	−0.08	−0.38**	−0.04	−0.15	−0.32**	0.38**
	Sex	0.11	−0.12	0.02	−0.01	−0.02	0.13
	Mood	−0.04	−0.20	−0.02	0.01	−0.06	0.06

*FL* first language (English/Not English), *YOE* years of education

^*^p < 0.05, **p < 0.001

Given that age, YOE, and FL were found to be correlated with performance on the Q-CAS, participants’ age and YOE were divided into tertiles using quantile bins to create groups of roughly equal size. Table 5 presents the normative data for the cognitive domains split by age groups, YOE groups separately by FL. The tenth percentile cutoff (z = −1.28) was chosen to ensure that more subtle cases of cognitive impairment are not missed. Normative data by subtests are included for completeness in the Supplemental Materials (see Table S2a,b).

**Table 5:** 10th Percentile cut-off in the normative sample by age, YOE, and FL

	Age	YOE	Memory	Language	Perception	Attention	Executive	Speed
EFL	18–42	< 13	18	5	6	5	10	58
		13–15	18	6	5	5	17	51
		16 +	19	5	5	6	16	51
	43–61	< 13	17	5	5	5	15	54
		13–15	17	5	5	5	14	50
		16 +	17	5	5	5	16	46
	62 +	< 13	17	4	5	5	12	63
		13–15	17	5	5	6	18	65
		16 +	17	5	5	6	18	50
ESL	18–42	< 13	18	2	5	4	9	47
		13–15	18	2	5	5	8	44
		16 +	19	3	5	5	13	42
	43–61	< 13	17	2	5	5	6	57
		13–15	17	1	5	3	9	61
		16 +	18	3	5	5	12	50
	62 +	< 13	17	2	5	3	6	67
		13–15	18	2	5	4	9	59
		16 +	17	3	5	6	11	51

*EFL* english first language, *ESL* english second language, *YOE* years of education

### Stroke sample

One hundred and fifty patients with acute stroke completed the Q-CAS. The demographic and clinical characteristics of the sample are described in Table 2. Most patients were above 60 years of age with 12–16 years of formal education. Most patients had a mild-to-moderate stroke based on the NIHSS, and there was a relatively equal proportion of patients with left- and right-hemisphere lesions. The average time patients took to complete the Q-CAS was 15 min with a range of 12–18 min. The performance of the patients is summarized in Table 3.

Most subtests had high completion rates, with fewer than 10% missing data for over half of the Q-CAS items. The subtests with the most missing data were Design fluency followed by Months Backwards and Canceling 2s. Although no systematic data were collected regarding reasons for non-completion, inspection of the raw data showed that a majority of patients attempted the subtests but either could not grasp the instructions due to cognitive impairment and/or language barrier, or had motor limitations affecting task completion. Importantly, as was embedded into the design of the Q-CAS, missing data rate across domains was very low, at 5% for Language and Processing Speed and < 1% for Attention and Executive Function. Thus, the Q-CAS was able to capture something about performance in all domains, for almost all patients, even if not all subtests were completed.

Correlation analyses showed that older age was associated with poorer memory, language, and speed; more education with better language, executive function, and speed; and English as a second language with poorer language, attention, executive function, and speed (see Table 4 and S1b for subtest correlations). Sex or mood, like in the healthy control, was not correlated with performance on any cognitive domains.

### Reliability

There were 31 healthy control participants who were retested on the Q-CAS (mean days between test–retest = 38, SD = 14, range = 21 to 63). The total score demonstrated moderate absolute agreement across testing occasions (ICC = 0.67). Subtest reliability varied from moderate to good (see Table S3). In contrast, poor reliability was observed for the perception subtest (ICC = −0.02), which appeared to be driven by a substantial ceiling effect. Most participants (26/31) scored at ceiling at both time points, with minimal variability among the remaining cases, resulting in restricted score variance and an attenuated ICC. Similarly, ICCs for Orientation, Recognition, and Spatial subtests could not be reliably estimated due to restricted score variability across participants.

### Convergent and divergent validity

All Q-CAS subtests showed convergent validity with standardized neuropsychological measures with highly significant p values across most subtests (see Table 6). All correlation coefficients were above the predetermined 0.30 cutoff. All Q-CAS subtests also showed divergent validity with a measure of lower limb strength with all correlation coefficients below the predetermined 0.30 cutoff (see Table S4).

**Table 6:** Convergent correlations between Q-CAS subtest and construct-matched neuropsychological tests

Cognitive domain	Q-CAS subtest	Convergent task	*n*	*r*/*ρ*	*p*
Memory	Orientation	Orientation questions (date, age, year, day, hospital name)	87	0.50	< 0.001
	Verbal recall	Verbal paired associates	67	0.46	< 0.001
	Verbal recognition	Verbal paired associates	67	0.34	0.006
	Spatial location recall	Location learning test	69	0.31	0.010
Language	Picture naming	rGNT/Oldfield	84	0.30	0.007
Perception	Incomplete numbers	VOSP incomplete letters	87	0.40	< 0.001
	Star counting	VOSP dot counting	85	0.53	< 0.001
Attention	Digit span (forward and backwards)	WAIS digit span (forward and backwards)	85	0.75	< 0.001
Executive function	Motor tapping	HSCT (Cat. A and B Errors)	66	0.31	0.013
	Phonemic fluency	Phonemic fluency	84	0.73	< 0.001
	Design fluency	D-KEFS design fluency test	57	0.51	< 0.001
Processing speed	Months backwards	SDMT	62	0.46	< 0.001
	Counting 3 s	Counting Bs	80	0.58	< 0.001
	Canceling 2 s	Canceling As	72	0.74	< 0.001
Mood	Depression score	PHQ-2	85	0.60	< 0.001
	Anxiety score	GAD-2	85	0.53	< 0.001

rGNT: revised Graded Naming Test; VOSP: Visual Object and Space Perception battery; WAIS: Wechslar Adult Intelligence Scale; HSCT: Hayling Sentence Completion Test; D-KEFS: Delis-Kaplan Executive Function System; SDMT: Symbol-Digit Modality Test; PHQ: Patient Health Questionnaire; GAD: Generalised Anxiety Disorder

### Diagnostic performance: ROC curve analysis

**Fig. 1:**
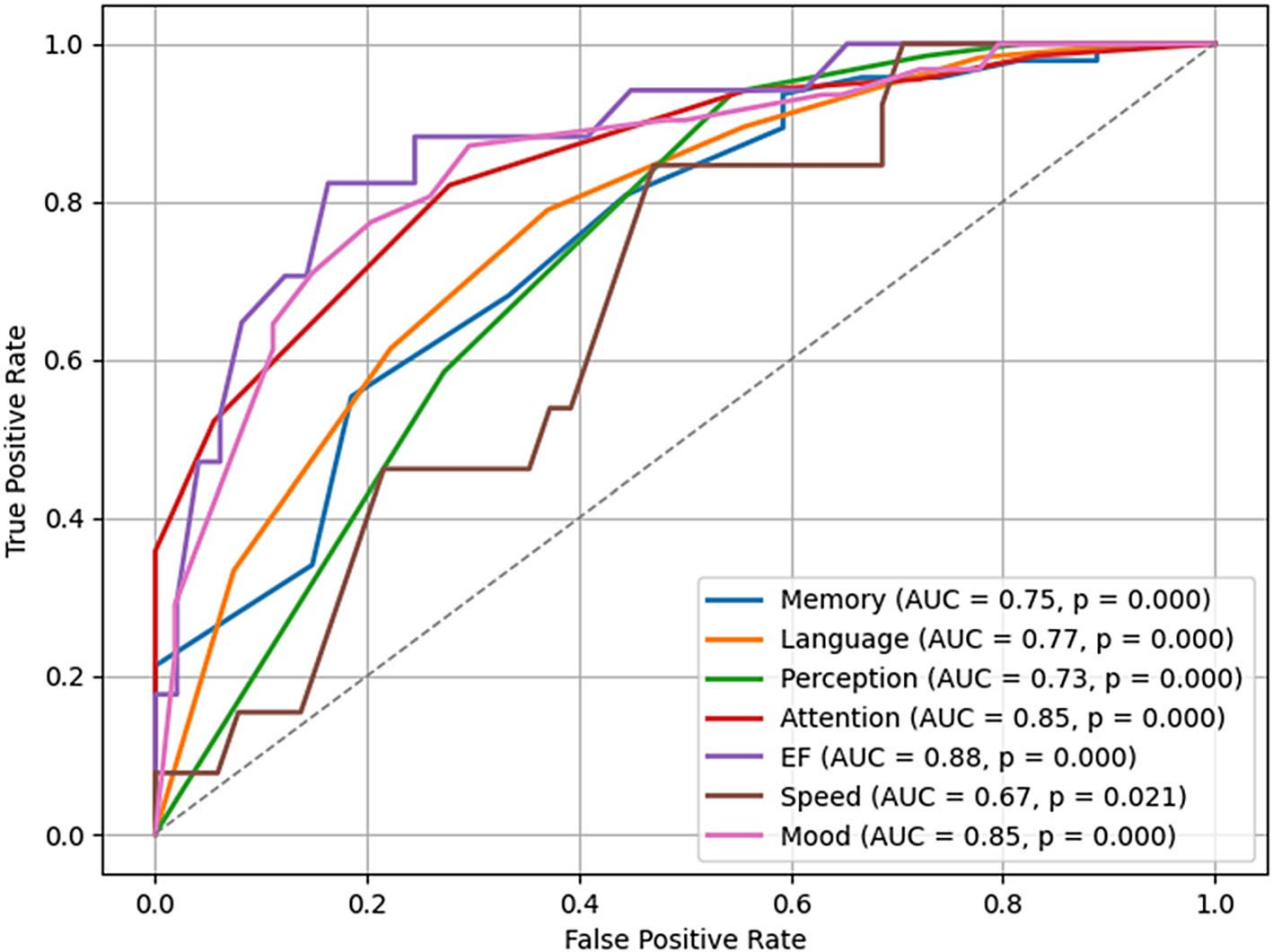
Receiver-operating characteristic (ROC) curves for the different Q-CAS domains

We examined the ability of Q-CAS domain scores to accurately identify true impairment in stroke patients as defined by reference-standard neuropsychological classification. ROC curve analyses demonstrated significant discrimination across all domains, with particularly excellent performance (AUC > 0.8) observed for Attention, Executive Functions, and Mood (see Fig. 1). Critically, the AUCs for all domains were statistically better than chance. We also conducted separate ROC curve analyses for patients with left- and right-hemisphere lesions to evaluate whether Q-CAS sensitivity varied as a function of lesion laterality. Across domains, bootstrapped comparisons of AUCs revealed no statistically significant differences between left- and right-hemisphere groups (all *p* > 0.05), indicating broadly comparable predictive performance across lesion laterality.

### Determining clinical cut-off for diagnostic predictions

Q-CAS subtest cut-offs at the 10th percentile were derived from the full normative sample (Table S5), with adjustments for age (Recall, Cancelling 2 s) and first language (Picture Naming, Phonemic Fluency). Domain impairment was classified as impaired performance on any subtest within that domain. Compared against reference-standard neuropsychology classification, Executive Function, Speed, and Memory showed highest sensitivity (> 0.8), Perception, and Attention showed highest specificity (> 0.8). Executive functions and Speed showed the highest positive predictive value (> 0.8), while Attention, Memory, and Perception showed the highest negative predictive value (> 0.8; see Table 7).

**Table 7:** Diagnostic accuracy of the Q-CAS

Domain	Sensitivity	Specificity	PPV	NPV
Memory	0.815	0.571	0.512	0.848
Language	0.556	0.737	0.5	0.778
Perception	0.455	0.938	0.714	0.836
Attention	0.722	0.821	0.52	0.917
Executive	0.875	0.571	0.862	0.6
Speed	0.821	0.6	0.902	0.429

### Determining clinical cut-off for mood

As reported above, mood did not significantly correlate with performance on any cognitive domains in the healthy control or patient group. Examining depression and anxiety scores separately also did not reveal any significant correlations. Based on the normative sample, the 10th percentile cut-off for mood was 6 for depression and 7 for anxiety. However, using the patient group data and comparing it against standardized measures of mood (PHQ-2 and GAD-2), a more conservative cut-off score of 4 on the new screening test provided the best balance between sensitivity and specificity. For depression, this cut-off yielded sensitivity = 0.83 and specificity = 0.66, and for anxiety, it resulted in sensitivity = 0.82 and specificity = 0.63.

## Discussion

The Q-CAS is a new cognitive screening tool that covers six cognitive domains and mood. It was designed to overcome the limitations of existing screening tools, which do not assess, or assess insufficiently, areas of non-verbal memory, executive functions, and processing speed. It also includes a simple measure of mood, which does not exist in any other cognitive screening tools. In this study, we presented normative data for the Q-CAS across a large range of ages and educational levels. The data were split according to whether English was the participant’s first language, so that appropriate norms could be applied when assessing non-native English speakers. We demonstrated that the Q-CAS is reliable and valid for use in an acute stroke population. It was sensitive to detecting post-stroke cognitive and mood impairment when compared with reference-standard neuropsychological assessment. A novel scoring approach was devised for the Q-CAS in order to take into consideration patient demographic factors where relevant, while maintaining brevity.

Construct validity was established for all Q-CAS subtests. Performance on Q-CAS subtests was significantly correlated with construct-matched neuropsychological measures, with correlation coefficients above the predefined ‘adequate’ threshold of 0.3 for all tests. Digit span, phonemic fluency, and canceling 2’s demonstrated ‘very good’ correlations (> 0.7), reflecting the fact that these subtests were direct adaptations of the corresponding neuropsychological measures. The relatively low, though statistically significant, correlation for the naming subtest is likely attributable to a ceiling effect in the screening measure among EFL patients, in contrast to the validation measure, the revised Graded Naming Test, which has a broader standard deviation [27]. This reflects a trade-off in developing a language subtest suitable for individuals with both EFL and ESL. All subtests also showed adequate divergent correlations with a measure of lower limb strength, confirming that the Q-CAS measures constructs  are distinct from post-stroke motor abilities. Together, these findings provide evidence that Q-CAS subtest performance can be validly interpreted in accordance with the relevant cognitive domains. This extends beyond the original validation of other commonly used cognitive screening tools, which typically assess only the validity of overall scores, such as the MoCA [34], or rely primarily on comparisons with items from other screening measures, as in the OCS [15] and OCS Plus [30]. Indeed, we have previously demonstrated poor correspondence between MoCA-defined cognitive domains and performance on comparable neuropsychological assessments [13]. In busy clinical practice, clinicians are often inclined to interpret performance on individual subtests because of their apparent face validity. Our findings provide important reassurance that such interpretation is justified when using the Q-CAS.

All Q-CAS cognitive domains and the mood screen demonstrated significant discrimination between impaired and unimpaired performance in stroke patients relative to gold-standard neuropsychological classification, as reflected by ROC curve analyses. Discrimination was particularly strong for attention, executive functions, and mood. Importantly, sensitivity was comparable in patients with left- and right-hemisphere lesions, suggesting that, unlike the MoCA [14], the Q-CAS is similarly sensitive to impairments associated with either hemisphere. Like the OCS, the Q-CAS was designed to be domain-specific, so that a patient’s particular strengths and weaknesses can be identified. However, while the OCS and Q-CAS overlap in their assessment of memory, attention, and language, the Q-CAS was also designed to capture perception, executive functions, processing speed, and mood—domains not assessed by the OCS. These cognitive and affective domains are frequently affected following stroke and have been shown to be important predictors of long-term functional outcomes [5]. Thus, accurate assessment of these domains, particularly in the early post-stroke period, should be used to inform rehabilitation planning and support targeted intervention.

To account for individual demographic factors, a novel scoring approach was developed for the Q-CAS. Without the use of machine learning algorithms, it is difficult to account for multiple demographic variables simultaneously without compromising the screen’s simplicity and clinical utility. In the MoCA, one point is added to the total score for individuals with more than 12 years of education; however, the psychometric rationale for this adjustment is not provided [34]. For the Q-CAS, we proposed a novel subtest-level scoring adjustment based on observed correlations with demographic variables in the normative sample. Notably, because construct validity was demonstrated at the subtest level, domain-level impairment was defined as impairment on any one subtest within a domain rather than using a more traditional cumulative cut-off score approach. This flexible design means that a clinician may still gain useful information about a patient’s functioning in a cognitive domain even if the patient is unable to complete all subtests. When these adjustments were applied, diagnostic accuracy across cognitive domains was acceptable, with particularly high sensitivity for memory, executive functions, and processing speed. This approach to administering and scoring the Q-CAS provides a simple and efficient means of understanding a patient’s domain-level cognitive functioning while allowing for adjustment based on demographic factors, and it can be implemented using only a single sheet of paper and a pen.

Alternative to this abbreviated scoring approach, clinicians may prefer to use the comprehensive normative table provided in this paper to classify domain impairment based on a patient’s particular age, years of education, and first language. For example, in cases where there is uncertainty regarding a patient’s domain-specific cognitive classification, perhaps if encountering a patient who is particularly young or old, or with particularly high education. Reference to the normative table may provide some insight and guidance into how to interpret their performance. However, it should be noted that the binning of continuous demographic variables always carries a caveat as the reduction in effective sample size across strata may introduce spurious effects that reduce the robustness of parameter estimates [35]. Future work would benefit from more recent machine learning approaches that are agnostic to predefined thresholds, as such methods could address some of the limitations inherent in the traditional normative adjustment techniques while preserving or enhancing diagnostic performance.

Sex and mood did not show any significant correlation with performance across the cognitive domains, either in the large normative sample or in the stroke sample. Findings regarding sex differences in neuropsychology are mixed, and imbued with historical, biological and social-cultural confounds [36]. When present, these differences tend to be small to moderate in effect size and appear to be task-dependent [37]. The absence of sex effects in the Q-CAS may reflect the limited sensitivity of these relatively brief and simplified tasks to detect subtle differences. Similarly, although low mood and anxiety are known to impact cognitive test performance, their effects may be too subtle to detect with short screening measures. Nevertheless, including an embedded mood measure within the Q-CAS ensures that mood can be routinely considered as part of the patient’s neuropsychological formulation, as recommended by the national and international guidelines [17]. Notably, the cut-off scores for possible mood difficulties were more conservative in the stroke sample when using standardized mood measures (cut-off 4/10) compared with the normative sample (cut-off 6–7/10). These findings suggest that even relatively low-level endorsements of mood and anxiety symptoms in stroke patients warrant further consideration.

Several limitations should be considered. While a concerted effort was made to recruit participants across a broad range of ages and educational levels, and to achieve relatively balanced groups across permutations of demographic variables, some subgroups were inevitably more difficult to recruit due to structural and sociocultural barriers, including limited access, lower likelihood of engagement with research, and challenges in reaching older adults. Furthermore, this was a single-site study, and although London is a cosmopolitan city with a diverse sociodemographic and cultural population, there may still be idiosyncrasies that limit the generalizability of the normative dataset. Now that the fundamental design and principles of the Q-CAS have been established, it will be important to cross-validate the measure in different populations. It should also be acknowledged that only patients who were able to provide informed consent were recruited, and therefore, the sample was largely restricted to individuals with mild-to-moderate strokes. Patients with more severe cognitive impairments, such as significant aphasia or perceptual impairments, were excluded. As such, the utility of the Q-CAS for patients with more severe strokes remains unclear. The Q-CAS was designed to detect more subtle deficits that might be missed, as individuals with more severe impairment are less likely to require a screening measure before being referred directly for specialist assessment.

In conclusion, the Q-CAS is a novel cognitive screening tool that has been validated in a stroke population. It offers several advantages over existing tools: it is brief and easy to administer, covers six important cognitive domains and mood, is sensitive to domain-specific cognitive impairment, and accounts for relevant demographic factors. While cognitive screening cannot replace the psychometric rigor of a comprehensive neuropsychological assessment, the Q-CAS provides a practical first-line approach that mitigates limitations of widely used tools, such as the MMSE, MoCA, and OCS, offering sensitive, domain-specific assessment across cognitive and affective domains with an effective balance between brevity and thoroughness.

## Supplementary Information

Below is the link to the electronic supplementary material.

Supplementary file1 (DOCX 40 KB)

## Data Availability

The datasets generated and/or analyzed during the current study are not publicly available due to privacy reasons but are available from the corresponding author on reasonable request.
