# Supplementary material for: The Queen Square cognitive assessment screen (Q-CAS): normative data and validation in acute stroke
Source: J Neurol. 2026 Jul 20;273(8):477. doi: 10.1007/s00415-026-14016-4 (PMC13385056; doi:10.1007/s00415-026-14016-4)
Supplement: Supplementary file 1 — Supplementary file1 (DOCX 40 KB) [file 415_2026_14016_MOESM1_ESM.docx]

**Supplemental Material**

**Table S1.** Correlation table showing Spearman’s rho coefficients between the Q-CAS subtest performance and demographic factors and mood for (a) healthy control participants and (b) patients with acute stroke

**(a)**

|  | **Memory** | **Recall** | **Recog** | **Spatial** | **Language** | **Perception** | **Attention** | **Executive Function** | **Tapping** | **Fluency** | **Design** | **Speed** | **Months** | **Count3** | **Cancel2** |
| --- | --- | --- | --- | --- | --- | --- | --- | --- | --- | --- | --- | --- | --- | --- | --- |
| **Age** | **-0.39**** | **-0.39**** | **-0.08*** | **-0.08*** | 0.04 | **-0.08*** | -0.05 | 0.01 | -0.06 | **0.1**** | **-0.09**** | **0.24**** | **0.1**** | **0.21**** | **0.41**** |
| **YOE** | **0.22**** | **0.22**** | 0.04 | 0.01 | -0.04 | 0.07 | **0.15**** | **0.19**** | **0.11*** | **0.15**** | **0.15**** | **-0.16**** | **-0.19**** | -0.04 | **-0.15**** |
| **FL** | 0.06 | 0.06 | -0.07 | -0.02 | **-0.59**** | -0.04 | **-0.16**** | **-0.28**** | 0.03 | **-0.33**** | **-0.12*** | -0.04 | 0 | -0.04 | **-0.09*** |
| **Sex** | 0.04 | 0.03 | 0.01 | 0.08 | -0.06 | 0 | 0.04 | -0.01 | 0.01 | -0.06 | 0.05 | -0.01 | **0.08*** | -0.07 | **-0.12**** |
| **Mood** | 0.05 | 0.05 | -0.04 | 0.03 | 0.04 | -0.05 | -0.04 | 0.02 | 0.03 | 0.01 | 0.05 | 0.04 | 0.04 | 0.05 | 0.02 |

**(b)**

|  | **Memory** | **Recall** | **Recog** | **Spatial** | **Language** | **Perception** | **Attention** | **Executive Function** | **Tapping** | **Fluency** | **Design** | **Speed** | **Months** | **Count3** | **Cancel2** |
| --- | --- | --- | --- | --- | --- | --- | --- | --- | --- | --- | --- | --- | --- | --- | --- |
| **Age** | **-0.25*** | **-0.30**** | -0.16 | -0.16 | **-0.26*** | -0.12 | 0.01 | -0.13 | -0.09 | -0.12 | -0.03 | **0.20*** | 0.00 | **0.18*** | **0.23*** |
| **YOE** | 0.15 | 0.07 | **0.18*** | **0.18*** | **0.26*** | 0.14 | **0.24*** | **0.37**** | **0.23*** | **0.29**** | **0.24*** | **-0.28*** | **-0.22*** | -0.13 | **-0.25*** |
| **FL** | -0.08 | 0.03 | -0.12 | -0.05 | **-0.38**** | -0.04 | -0.15 | **-0.32**** | **-0.19*** | **-0.33**** | -0.15 | **0.38**** | **0.26*** | **0.25*** | **0.33**** |
| **Sex** | -0.04 | -0.14 | -0.15 | -0.06 | -0.20 | -0.02 | 0.01 | -0.06 | -0.05 | -0.10 | 0.09 | 0.06 | 0.13 | 0.08 | 0.00 |
| **Mood** | 0.11 | 0.14 | -0.07 | 0.07 | -0.12 | 0.02 | -0.01 | -0.02 | 0.09 | -0.09 | 0.01 | 0.13 | 0.15 | 0.05 | 0.05 |

**Table S2** 10^th^ Percentile cut-off in the normative sample by Age and YOE for (a) those whose English is their first language and (b) English is their second language.

1. **English as first language**

| **Age** | **18–42** | | | **43–61** | | | **62+** | | |
| --- | --- | --- | --- | --- | --- | --- | --- | --- | --- |
| **YOE** | **<13** | **13–15** | **16+** | **<13** | **13–15** | **16+** | **<13** | **13–15** | **16+** |
| **Memory** | 18 | 18 | 19 | 17 | 17 | 17 | 17 | 17 | 17 |
| Orientation | 3 | 3 | 3 | 3 | 3 | 3 | 3 | 3 | 3 |
| Recall | 3 | 2.7 | 4 | 2 | 3 | 2 | 2 | 2 | 2 |
| Recognition | 6 | 6 | 6 | 6 | 6 | 6 | 6 | 6 | 6 |
| Spatial | 6 | 6 | 6 | 6 | 6 | 6 | 6 | 6 | 6 |
| **Language** | 5 | 6 | 5 | 5 | 5 | 5 | 4 | 5 | 5 |
| **Perception** | 6 | 5 | 5 | 5 | 5 | 5 | 5 | 5 | 5 |
| **Attention** | 5 | 5 | 5 | 5 | 4 | 5 | 5 | 6 | 6 |
| **Executive Function** | 10 | 17 | 16 | 15 | 14 | 16 | 12 | 18 | 18 |
| Tapping | 3 | 3 | 3 | 3 | 3 | 3 | 2 | 3 | 3 |
| Fluency | 3 | 4 | 6 | 5 | 6 | 6 | 5 | 7 | 7 |
| Design | 6 | 5 | 6 | 5 | 2 | 7 | 0 | 3 | 6 |
| **Speed** | 58 | 51 | 51 | 54 | 50 | 46 | 63 | 65 | 50 |
| Months | 28 | 22 | 26 | 23 | 23 | 20 | 25 | 24 | 19 |
| Count3 | 17 | 14 | 16 | 16 | 15 | 15 | 21 | 19 | 18 |
| Cancel2 | 17 | 16 | 15 | 16 | 18 | 15 | 20 | 19 | 19 |

1. **English as second language**

| **Age** | **18–42** | | | **43–61** | | | **62+** | | |
| --- | --- | --- | --- | --- | --- | --- | --- | --- | --- |
| **YOE** | **<13** | **13–15** | **16+** | **<13** | **13–15** | **16+** | **<13** | **13–15** | **16+** |
| **Memory** | 18 | 18 | 19 | 17 | 17 | 18 | 17 | 18 | 17 |
| Orientation | 3 | 3 | 3 | 3 | 3 | 3 | 3 | 3 | 3 |
| Recall | 3 | 3 | 4 | 1 | 3 | 3 | 2 | 3 | 2 |
| Recognition | 6 | 6 | 6 | 6 | 6 | 6 | 6 | 6 | 6 |
| Spatial | 6 | 6 | 6 | 6 | 6 | 6 | 6 | 6 | 6 |
| **Language** | 2 | 2 | 3 | 2 | 1 | 3 | 2 | 2 | 2 |
| **Perception** | 5 | 5 | 5 | 5 | 5 | 5 | 5 | 5 | 5 |
| **Attention** | 4 | 5 | 5 | 4 | 3 | 5 | 3 | 4 | 5 |
| **Executive Function** | 9 | 8 | 13 | 6 | 9 | 12 | 6 | 9 | 11 |
| Tapping | 2 | 3 | 3 | 3 | 3 | 3 | 3 | 3 | 3 |
| Fluency | 2 | 2 | 4 | 1 | 2 | 3 | 3 | 3 | 4 |
| Design | 2 | 2 | 5 | 1 | 1 | 5 | -1 | 2 | 4 |
| **Speed** | 47 | 44 | 42 | 57 | 61 | 50 | 67 | 59 | 51 |
| Months | 24 | 18 | 19 | 21 | 31 | 18 | 29 | 26 | 21 |
| Count3 | 14 | 13 | 14 | 18 | 18 | 18 | 17 | 21 | 19 |
| Cancel2 | 13 | 15 | 15 | 23 | 17 | 16 | 18 | 20 | 18 |

**Table S3.** Test-retest absolute-agreement intraclass correlation coefficients (ICC) and p-values for Q-CAS subtests and Total score.

| **Cognitive domain** | **Q-CAS subtest** | **ICC** | **p-value** |
| --- | --- | --- | --- |
| **Memory** | Orientation | - | - |
|  | Verbal Recall | 0.43 | 0.001 |
|  | Verbal Recognition | - | - |
|  | Spatial Location Recall | - | - |
| **Language** | Picture Naming | 0.68 | <0.001 |
| **Perception** | Incomplete Numbers and | -0.02 | 0.531 |
|  | Star Counting |  |  |
| **Attention** | Digit span (Forward and Backwards) | 0.73 | <0.001 |
| **Executive Function** | Motor Tapping | 0.45 | 0.004 |
|  | Phonemic Fluency | 0.74 | <0.001 |
|  | Design Fluency | 0.70 | <0.000 |
| **Processing Speed** | Months Backwards | 0.54 | 0.001 |
|  | Counting 3s | 0.62 | <0.000 |
|  | Cancelling 2s | 0.65 | <0.000 |
| **Mood** | Depression and Anxiety | 0.66 | <0.000 |
| **Total Score** | - | 0.67 | <0.000 |

**Table S4.** Divergent correlations for each Q-CAS subtest.

| **Cognitive domain** | **Q-CAS subtest** | **Divergent task** | **n** | **r / ρ** | **p** |
| --- | --- | --- | --- | --- | --- |
| **Memory** | Orientation | Lower Limb Strength | 135 | 0.06 | 0.481 |
|  | Verbal Recall | Lower Limb Strength | 121 | 0.15 | 0.092 |
|  | Verbal Recognition | Lower Limb Strength | 121 | 0.22 | 0.016 |
|  | Spatial Location Recall | Lower Limb Strength | 123 | 0.06 | 0.499 |
| **Language** | Picture Naming | Lower Limb Strength | 128 | 0.19 | 0.032 |
| **Perception** | Incomplete Numbers | Lower Limb Strength | 134 | 0.05 | 0.591 |
|  | Star Counting | Lower Limb Strength | 134 | 0.00 | 0.962 |
| **Attention** | Digit span (Forward and Backwards) | Lower Limb Strength | 134 | 0.16 | 0.065 |
| **Executive Function** | Motor Tapping | Lower Limb Strength | 131 | 0.10 | 0.249 |
|  | Phonemic Fluency | Lower Limb Strength | 130 | 0.18 | 0.036 |
|  | Design Fluency | Lower Limb Strength | 102 | 0.28 | 0.004 |
| **Processing Speed** | Months Backwards | Lower Limb Strength | 108 | -0.09 | 0.348 |
|  | Counting 3s | Lower Limb Strength | 124 | 0.17 | 0.067 |
|  | Cancelling 2s | Lower Limb Strength | 109 | 0.18 | 0.058 |
| **Mood** | Depression Score | Lower Limb Strength | 84 | 0.02 | 0.886 |
|  | Anxiety Score | Lower Limb Strength | 84 | -0.10 | 0.370 |

**Table S5.** Cut-off scores by Q-CAS subtest used for the abbreviated cut-off approach.

| **Cognitive domain** | **Q-CAS subtest** | **Cut-off Score** |
| --- | --- | --- |
| **Memory** | Orientation | 3 |
|  | Verbal Recall | Age <=37: 3, >37:2 |
|  | Verbal Recognition | 6 |
|  | Spatial Location Recall | 6 |
| **Language** | Picture Naming | FL English: 5, Other: 2 |
| **Perception** | Incomplete Numbers and Star Counting | 5 |
| **Attention** | Digit span Forwards and Backwards | 5 |
| **Executive Function** | Motor Tapping | 3 |
|  | Phonemic Fluency | FL English: 5, Other: 2 |
|  | Design Fluency | 4 |
| **Processing Speed** | Months Backwards | 23 |
|  | Counting 3s | 17 |
|  | Cancelling 2s | Age <=52: 15, >52:19 |
